# Melatonin Synergises the Chemotherapeutic Effect of Temozolomide in Glioblastoma by Suppressing NF‐κB/COX‐2 Signalling Pathways

**DOI:** 10.1111/jcmm.70778

**Published:** 2025-08-13

**Authors:** Hong Tang, Qi Dai, Ziyan Zhao, Weiye Ge, Danlei Li, Zelin Chang, Penglai Pi, Jia Li, Zheng Sun

**Affiliations:** ^1^ Department of Integrated Medicine, College of Integrated Medicine Dalian Medical University Dalian People's Republic of China; ^2^ The First Affiliated Hospital of Dalian Medical University Dalian People's Republic of China

**Keywords:** glioblastoma, melatonin, NF‐κB/COX‐2, temozolomide

## Abstract

Glioblastoma (GBM) is an aggressive and highly malignant primary brain tumour, accounting for a significant proportion of adult brain tumours. It is associated with a poor prognosis and high recurrence rates. Although temozolomide (TMZ) remains the standard first‐line chemotherapy for GBM, its clinical efficacy is often limited by the development of drug resistance and toxic effects on normal tissues. Melatonin (Mel), a natural indoleamine synthesised by the pineal gland, has demonstrated synergistic anti‐tumour effects when combined with various chemotherapy agents in multiple studies. This study investigates the synergistic potential of Mel to enhance TMZ's therapeutic efficacy against GBM. The results demonstrate that the combination of Mel and TMZ significantly inhibits glioblastoma cell proliferation, migration, and invasion. Mechanistically, this synergistic effect is mediated through the NF‐κB/COX‐2 signalling pathway. Mel enhances TMZ's anti‐tumour activity by inhibiting IκBα phosphorylation, suppressing NF‐κB activation, and downregulating COX‐2 expression. Additionally, the combination treatment induced apoptosis via activation of the Caspase‐3 pathway. These results suggest that Mel can potentiate the therapeutic efficacy of TMZ in glioblastoma treatment, offering a promising strategy to overcome TMZ resistance while reducing its associated toxicity.

## 1 Introduction

Glioblastoma is a highly malignant primary brain tumour originating from neuroglial cells. It accounts for about 60% of all brain tumours in adults and 49% of malignant brain tumours [1, 2]. This type of tumour is known for its high malignancy, poor prognosis and aggressive invasiveness, often rapidly infiltrating surrounding brain tissue and causing severe neurological impairment [3, 4]. Temozolomide (TMZ) is the first‐line chemotherapy drug for glioblastoma. It is rapidly absorbed and effectively crosses the blood‐brain barrier, directly targeting tumour cells in the brain [5]. However, TMZ is known to be toxic to normal cells, potentially causing side effects such as gastrointestinal diseases (nausea, vomiting, and loss of appetite), haematological diseases (aplastic anaemia and bone marrow suppression), acute liver injury and other toxic effects [6, 7, 8, 9]. Additionally, TMZ resistance can arise due to factors such as the overactivation of DNA repair mechanisms in cancer cells, mutations in DNA damage repair signalling pathways, alterations in DNA repair signalling or overexpression of efflux transporters at the blood–brain tumour barrier [10, 11]. Therefore, combining TMZ with other therapeutic agents may improve its effectiveness by increasing cancer cells sensitivity to chemotherapy while minimising toxicity to normal cells [12, 13, 14].

N‐acetyl‐5‐methoxytryptamine (Mel) is an indoleamine compound produced by the pineal gland, known for its wide range of biological activities, including antioxidant, immunomodulatory, anti‐aging and tumour‐suppressing properties [15]. Recently, Mel's potential as an anti‐tumour agent has attracted growing attention [16, 17]. Research has shown that Mel can inhibit tumour cell growth and increase the sensitivity of cancer cells to chemotherapy drugs [18, 19, 20]. Additionally, studies have highlighted its synergistic effects in the treatment of various malignant tumours, including melanomas, ovarian cancer, colorectal cancer and aneurysms [21, 22, 23, 24]. Specifically, the combination of Mel with 5‐fluorouracil (5‐FU) has been shown to work through multiple mechanisms [25, 26], such as promoting apoptosis by enhancing oxidative activity, regulating the PI3K/Akt and Erk signalling pathways to induce cell death, stimulating MT3 receptors for neuroprotective and antitumour effects, regulating cancer stem cells, and modulating immune responses [27]. A meta‐analysis of randomised trials has demonstrated that Mel improves the efficacy of chemotherapy. When combined with chemotherapy agents like cisplatin, etoposide, fluorouracil and irinotecan, Mel significantly boosts treatment outcomes, including higher rates of complete remission, partial remission and stable disease. These results underscore Mel's potential to enhance the effectiveness of chemotherapy in treating various solid tumours by making cancer cells more responsive to treatment and possibly reducing the side effects associated with these therapies [28]. However, there is limited evidence regarding whether Mel can improve the therapeutic efficacy of TMZ in the treatment of glioblastoma.

Numerous studies have established that cyclooxygenase‐2 (COX‐2) plays a critical role in inflammation and can be induced by various stimuli, including growth factors and cytokines. This induction links COX‐2 to the progression of several human cancers [29, 30]. Previous research has shown that COX‐2 is highly expressed in various human tumours, including glioblastoma and that its expression is associated with increased tumour invasiveness and poor prognosis in patients [31, 32]. Specifically, COX‐2 levels have been found to rise significantly in the glioblastoma cell line T98G when treated with TMZ. Moreover, the combined use of COX‐2 inhibitors with TMZ effectively counters TMZ's impact on tumour spheroid growth and macrophage infiltration. These findings suggest that inhibiting COX‐2 expression may help suppress the development of human tumours [33, 34]. However, it remains unclear whether Mel, when combined with TMZ, can target COX‐2 expression and further inhibit glioblastoma cell growth. In this study, to address this confusion, we examined the combined impact of Mel and TMZ on glioblastoma cell proliferation, migration and apoptosis. Additionally, we employed network pharmacology and bioinformatics to predict the key target pathways and identify protein alterations to reveal the underlying molecular mechanisms. Our findings suggest that Mel enhances the anti‐tumour effect of TMZ, and this enhancement is mediated through the NF‐κB/COX‐2 signalling pathway. This indicates that the combination of Mel and TMZ could serve as an effective therapeutic strategy for glioblastoma.

## 2 Material and Methods

### 2.1 Experiment In Vitro

#### 2.1.1 Cell Lines and Cell Cultures

The human glioblastoma cell lines U87‐MG and U118‐MG, as well as the murine glioma cell line GL261, were obtained from the Cell Bank of the Chinese Academy of Sciences. All cell lines were cultured in DMEM supplemented with 10% fetal bovine serum (FBS) under standard conditions (37°C, 5% CO2).

#### 2.1.2 Reagents and Antibodies

TMZ was purchased from Coolaber (Beijing, China), Mel from MedChemExpress (MCE, Shanghai, China) and celecoxib (CB) from Aladdin (Shanghai, China). All compounds were initially dissolved in DMSO and then diluted in complete culture medium for use. Primary antibodies against BAX, Bcl‐2 and cleaved‐caspase 3 were obtained from Proteintech (Wuhan, China). Antibodies against COX‐2, IκBα, and phospho‐IκBα (p‐IκBα) were purchased from Cell Signalling Technology (Beverly, MA, USA). Antibodies against Cyt‐C and p65 were provided by Abclonal (Wuhan, China), and antibodies against caspase‐9 and β‐actin were obtained from Abclone (Wuhan, China). β‐Tubulin was purchased from Immunoway (San Jose, California, USA).

#### 2.1.3 Cell Viability Assay

Cell viability was assessed using the MTT assay (Roche Diagnostics, Indianapolis, IN, USA). U87‐MG and U118‐MG cells were seeded into 96‐well plates at a density of 5 × 10^3^ cells per well. For GL261 cells, the seeding density was 8 × 10^3^ cells per well in 96‐well plates. After overnight incubation, cells were treated with fresh medium containing varying concentrations of TMZ, Mel, or CB and incubated for 48 h. Following cell attachment, fresh medium with different concentrations of temozolomide (TMZ) and melatonin (Mel, dissolved in dimethyl sulfoxide DMSO, final DMSO concentration ≤ 0.1%) was added and incubated for 48 h. Absorbance was measured at 570 nm to evaluate cell viability. All treatments were performed in triplicate. Cell viability was expressed as a percentage relative to the melatonin 500 μM group.

#### 2.1.4 Drug Interaction Assessment

To assess whether Mel enhances the effect of TMZ, the Chou–Talalay method was used using CalcuSyn software (version 2, Biosoft, Cambridge, UK) to calculate the combination index (CI). The fraction affected (FA) was used to represent the percentage of affected cells, calculated as the inverse of cell viability (0%–100%). Interactions were classified as synergistic (CI < 1), antagonistic (CI > 1) or additive (CI = 1). CI values were calculated for TMZ and Mel both individually and in combination.

#### 2.1.5 Experiments of Colony Formation

To assess the effect of TMZ and Mel on colony formation, cells were treated with TMZ, Mel, or their combinations. After 48 h, the cells were harvested, counted, and seeded at a density of 1 × 10^3^ cells per well in six‐well plates. The cells were incubated for 7–10 days, and colonies were stained with 0.1% crystal violet for 15 min at room temperature (RT). Colonies with more than 80 cells were counted and photographed.

#### 2.1.6 Wound Healing Assay

Cell migration was measured using a wound‐healing assay. Cells were grown to 80%–90% confluence in six‐well plates and incubated overnight in a starvation medium. A uniform wound was created using a sterile 100 μL pipette tip, and detached cells were removed by washing with starvation medium. Cells were then treated with TMZ, Mel, or their combination and incubated. After 48 h, the wound gaps were observed and photographed, and the gap area was measured.

#### 2.1.7 Cell Invasion Assay

Cell invasion assays were performed using matrix‐coated transwell chambers (8.0 μm pores). Cells were grown overnight in serum‐free DMEM, resuspended, and added to the upper chamber (1 × 10^5^ cells/well) coated with Matrigel. The lower chamber contained DMEM with 10% FBS. After 48 h of TMZ treatment, Mel, or their combination, non‐invading cells were removed, and invading cells were stained with 0.1% crystal violet. The number of invading cells was counted in three random microscopic fields.

#### 2.1.8 Western Blot Analysis

Protein lysates were separated using a 10% one‐step PAGE gel preparation kit (Sparkjade, EC0023‐B, Shandong, China), transferred to a polyvinylidene difluoride (PVDF) membrane, and subjected to immunoblotting with specific antibodies. Protein bands were visualised by enhanced chemiluminescence.

#### 2.1.9 Measurement of Mitochondrial Membrane Potential

Mitochondrial membrane potential (Δψm) was evaluated using the JC‐1 fluorescent probe (Beyotime, Shanghai, China), following the manufacturer's instructions. Briefly, U118MG cells were harvested after treatment, washed twice with pre‐chilled PBS, and incubated in a mixture of 500 μL culture medium and 500 μL JC‐1 staining solution at 37°C in the dark. After incubation, cells were washed three times with cold buffer to remove excess dye and then imaged under a fluorescence microscope. JC‐1 forms red‐fluorescent aggregates in mitochondria with intact membrane potential, whereas it remains in a green‐fluorescent monomeric form in depolarised mitochondria. The red‐to‐green fluorescence intensity ratio was used as an indirect indicator of Δψm. Cells treated with CCCP (carbonyl cyanide 3‐chlorophenyl‐hydrazone) served as the positive control for Δψm disruption.

### 2.2 The Potential Analysis of Mel and TMZ Against Glioblastoma

#### 2.2.1 Target Prediction and Data Collection

Potential targets for ‘Melatonin’ and ‘Temozolomide’ were obtained from the Pubchem compound database (https://www.ncbi.nlm.nih.dov/). The results were complied and duplicates were removed to create a target database for both compounds. Glioblastoma‐related targets were obtained from several resources, including TTD (https://db.idrblab.net/ttd/), GeneCards (https://www.genecards.org/) and Drugbank (https://go.drugbank.com/). After merging the data and removing duplicates, glioblastoma‐specific targets were identified.

#### 2.2.2 Network Construction and Analysis

The Venny 2.1 online tool (https://bioinfogp.cnb.csic.es/tools/venny/) was used to identify shared targets between Mel, TMZ and glioblastoma. A Venn diagram was generated to visualise these common targets. The identified targets were then uploaded to the STRING 12.0 platform (https://string‐db.org/), with species set to ‘ *Homo sapiens* ’ and a minimum interaction score of 0.9. Unconnected nodes were removed, and the resulting data were exported in TSV format for network construction in Cytoscape 3.9.0. The CytoHubba plugin was explored to rank the top ten hub genes based on their DMNC scores, indicating their centrality in the network.

#### 2.2.3 Gene Ontology (GO) and Kyoto Encyclopedia of Genes and Genomes (KEGG) Pathway Enrichment Analysis

DAVID is a bioinformatics database that integrates biological data with analytical tools, offering comprehensive functional annotations for large‐scale gene or protein lists. It enables users to extract biological insights through GO and KEGG pathway analyses. The overlapping targets of Mel, TMZ and glioblastoma were input into the DAVID 6.8 database for GO and KEGG pathway enrichment analysis. The results were then visualised using the ‘ggplot2’ package in RStudio.

#### 2.2.4 Molecular Docking

Molecular docking is an efficient technique for exploring interactions between small molecules and their target proteins. To further analyze how Mel enhances the efficacy of TMZ against glioblastoma, PTGS2 (COX‐2) was selected for molecular docking with Mel and TMZ based on their DMNC scores. The crystal structure of COX‐2 (PDB ID: 5IKQ) was obtained from the PDB database, while the 3D structures of Mel and TMZ were retrieved from the PubChem database. These structures were then energy‐minimised using the MMFF94 force field.

Molecular docking was conducted using AutoDock Vina 1.1.2. Initially, aspirin was docked into the known binding pocket of COX‐2, followed by docking of TMZ and Mel. The binding pocket was set to 90 × 90 × 90 cubic angstroms volume to encompass all potential active sites. Receptor Protein Preparations included using PyMol 2.5.2 to remove water molecules, salts and small molecules. ADFR suite 1.0 was then used to convert both small molecules and proteins into PDBQT format, which is necessary for molecular docking. In this process, small molecules were treated as flexible, while the protein remained rigid. A global search detail of 32 was used, with an iterated local search global optimiser algorithm. The highest docking score indicated the optimal binding conformation. Finally, PyMol 2.5.2 was used to visualise and analyse the docking results for the ternary complex.

### 2.3 Experiment In Vivo

#### 2.3.1 Animal Experiments

All animal procedures were conducted in accordance with the NIH Guide for the Care and Use of Laboratory Animals and were approved by the Animal Ethics Committee of Dalian Medical University (Approval No. AEE22050). A total of 36 female C57BL/6 mice (4–6 weeks old, 14–18 g) were obtained from the SPF Animal Center of Dalian Medical University (Dalian, China) and randomly assigned to experimental groups. GL261 cells (2.5 × 10^6^ cells per mouse) were suspended in 150 μL PBS and subcutaneously injected into the mice. Tumour volume was measured every other day using callipers and calculated as *V* = (length × width^2^)/2. Once tumours reached approximately 50 mm^3^, mice were randomly divided into the following treatment groups (*n* = 6): (i) C group (vehicle control); (ii) M group (Mel, 100 mg/kg, i.p.); (iii) T group (TMZ, 15 mg/kg, i.p.); (iv) CB group (celecoxib, 15 mg/kg, i.p.); (v) MT group (Mel + TMZ, 100 mg/kg + 15 mg/kg, i.p.); (vi) CT group (celecoxib + TMZ, 15 mg/kg + 15 mg/kg, i.p.). After 8 days of treatment, mice were euthanised under isoflurane anaesthesia, and tumour tissues were harvested for further analysis.

#### 2.3.2 Haematoxylin and Eosin (H&E) Staining

Tumour tissues were fixed, embedded in paraffin and sectioned. Slides were dewaxed in xylene I and II, then rehydrated through a graded ethanol series (100%, 95%, 85%, 70%, 10 min each). Haematoxylin staining was carried out for 5 min, followed by bluing under running tap water and eosin staining for 2 min. After washing, slides were dehydrated through an ascending ethanol series (70%, 85%, 95%, 100%) and cleared in xylene before mounting with neutral resin. Stained sections were examined and photographed under a light microscope (Olympus, Tokyo, Japan).

#### 2.3.3 Immunohistochemical (IHC) Analysis

Human glioma and normal brain tissue samples were collected from the Department of Neurosurgery, the First Affiliated Hospital of Dalian Medical University (Ethical approval number: PJ‐KS‐KY‐2022‐26). Samples were fixed in 10% formalin and embedded in paraffin. IHC staining was performed using a commercial detection kit (ZSGB‐Bio, Beijing, China). COX‐2 antibody (1:200 dilution) was used for staining human samples. For xenografted mouse tumours, paraffin‐embedded sections were stained with antibodies against COX‐2 (1:200), Cyt‐C (1:1000), and p65 (1:1000). Positive staining was indicated by brown coloration in the cytoplasm or nucleus under a light microscope (Olympus, Tokyo, Japan). Expression levels were quantified using Image‐Pro Plus software.

### 2.4 Statistical Analysis

All the experiments were performed in triplicate. Data are presented as means ± standard deviations. Statistical analyses were performed using GraphPad Prism software. One‐way analysis of variance (ANOVA) and Student's *t*‐test were applied, with a *p*‐value < 0.05 considered statistically significant.

## 3 Results

### 3.1 Mel and TMZ Combination Enhanced Inhibition of Cell Viability and Proliferation

**FIGURE 1:**
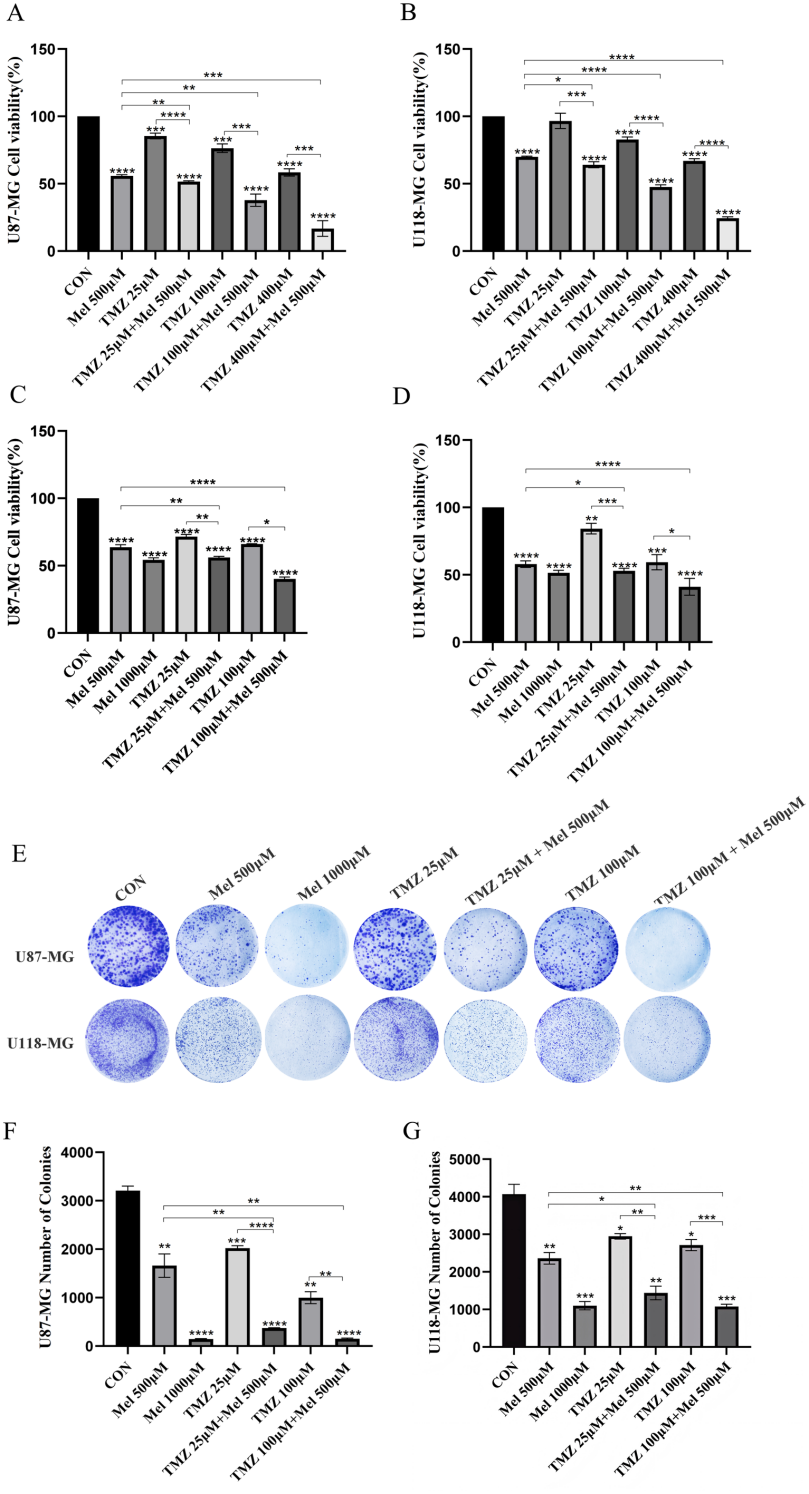
Effect of Mel and TMZ combination on cell viability and proliferation in GBM cells. (A, B) Human U87‐MG and U118‐MG cells were treated with Mel or TMZ or their combination at the indicated doses. At 48 h, the cell viability was measured using the MTT assay. Cell viability in the Mel 500 μM group was set as 100% reference. (C, D) U87‐MG and U118‐MG cells were treated with TMZ (25 μmol/L, 100 μmol/L) or Mel (500 μmol/L, 1000 μmol/L) alone or in combination. Colony formation of the GBM cells was captured in photographs (E), and the relative colony numbers were calculated (F, G). Data are presented as mean ± SD from three independent experiments. Statistical significance is indicated by *p < 0.05, **p < 0.01, ***p < 0.001, ****p < 0.0001.

**FIGURE 2:**
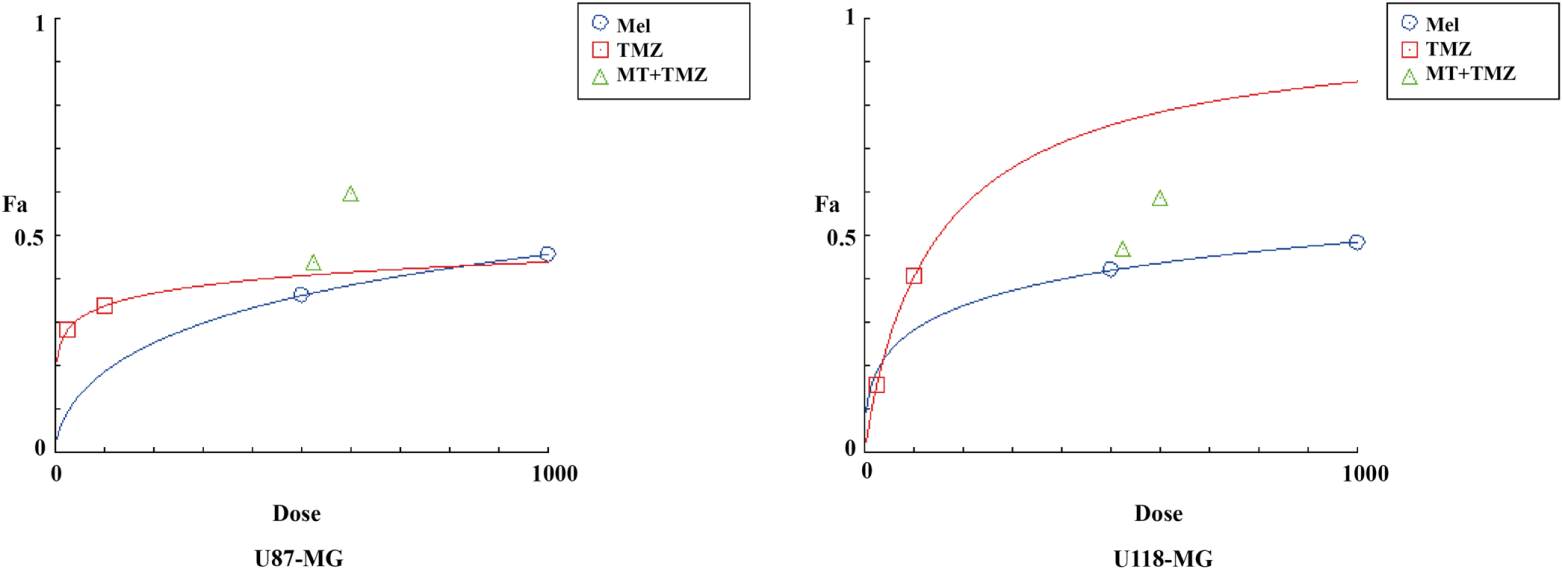
Dose‐response curve for the individual drugs and combination of Mel and TMZ.

To evaluate whether Mel can potentiate the inhibitory effects of TMZ on glioblastoma cell proliferation, we first examined the impact of Mel in combination with TMZ on the growth of human glioblastoma cell lines U87‐MG and U118‐MG (Figure 1A,B), as well as the murine glioblastoma cell line GL261 (Figure S1). After 48 h treatment with 500 μM Mel and varying concentrations of TMZ (25, 100, or 400 μM), cell viability was significantly reduced compared to treatment with either agent alone (*p* < 0.05). These findings demonstrate that the combination of Mel and TMZ exerts a markedly stronger inhibitory effect on cell viability in a dose‐dependent manner. To determine whether Mel works synergistically with TMZ, U87‐MG and U118‐MG cells were treated with the drugs either alone or in combination for 48 h. The combination index (CI) values for 500 μM Mel combined with 25 or 100 μM TMZ in both U87‐MG and U118‐MG cells were < 1, indicating a synergistic effect of Mel with TMZ against these glioblastoma cells (Figure 1C,D, Table 1). Using the Compusyn software, dose–response curves for the U87‐MG and U118‐MG cell lines were constructed (Figure 2). It was found that, in the U87‐MG cell line, the combination of Mel and TMZ resulted in a higher inhibition rate at equivalent doses compared to TMZ alone. However, in the U118‐MG cell line, the combination therapy was less effective than TMZ monotherapy. In a separate study by our research group examining the differential sensitivity of glioblastoma cell lines to Mel, proteomic analysis revealed that U87‐MG cells were more sensitive to Mel than U118‐MG cells, which is consistent with the findings of this study. We further examined the effect of the Mel–TMZ combination on the colony‐forming ability of glioblastoma cells using colony formation assays. As shown in Figure 1E, the combination treatment significantly inhibited colony formation in both U87‐MG and U118‐MG cell lines compared to the individual treatments. The difference was statistically significant (Figure 1F,G).

**TABLE 1:** The Fa–CI plots showed combination index (CI) in every fractional effect.

Cell line	Total dose	Fa	CI
	Mel (μM) + TMZ (μM)		
U87‐MG	500 + 25	0.439	0.59465
	500 + 100	0.598	0.18486
U118‐MG	500 + 25	0.47	0.7823
	500 + 100	0.589	0.62206

*Note:* Calculations were performed using CompuSyn software. CI values were determined for both individual Mel/TMZ treatments and the combined treatment, with CI values < 1 across the tested drug concentration range.

### 3.2 Migration and Invasion

**FIGURE 3:**
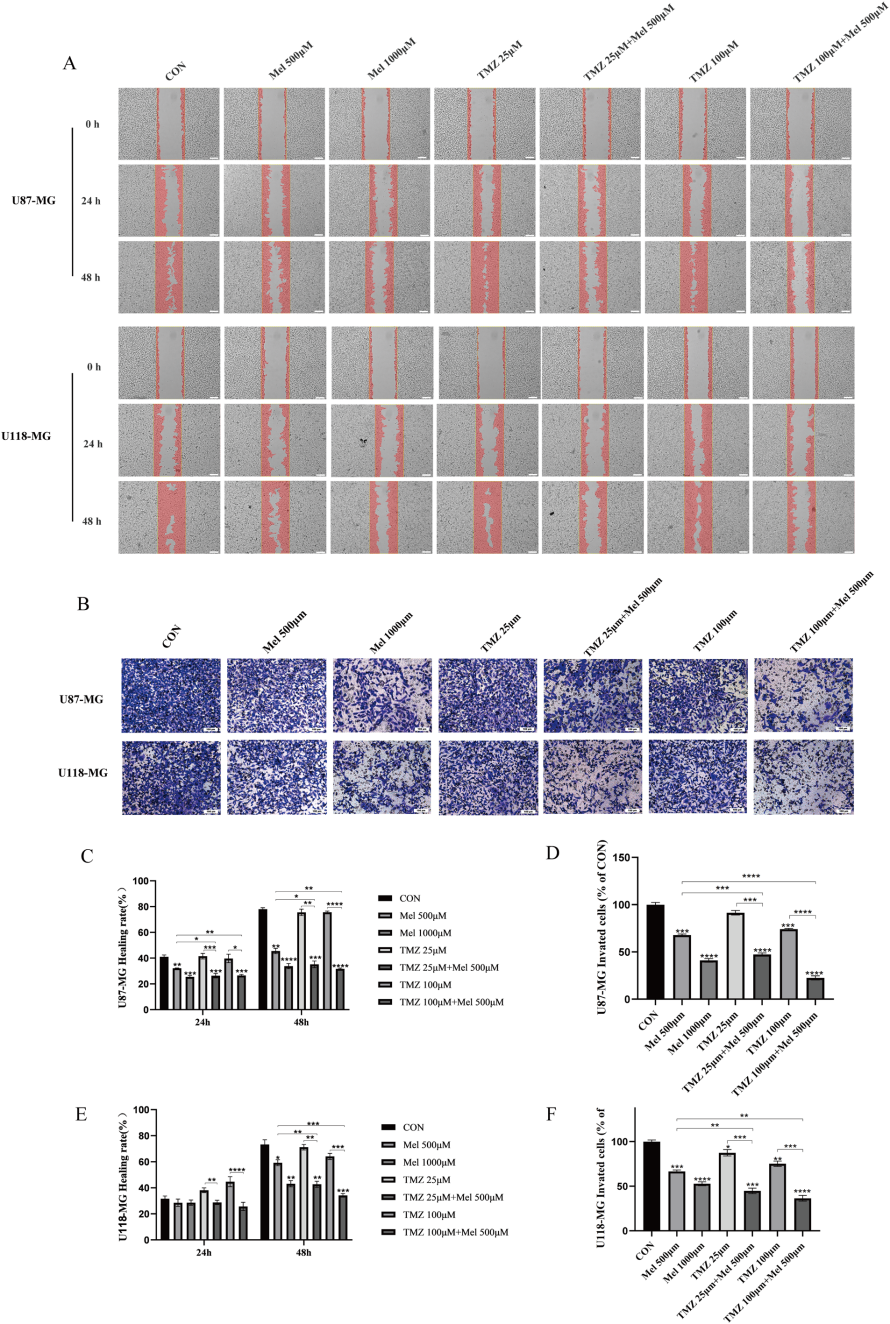
Effect of Mel and TMZ combination on cell migration and invasion in GBM cells. (A) Cell migration was analysed by a wound‐healing assay. The U87‐MG and U118‐MG cells were grown to full confluency, and the cell monolayers were wounded with a sterile pipette tip and washed with medium to remove detached cells from the plates (magnification 4×). Cells were then left either untreated or treated with Mel or TMZ alone or their combination. After 48 h, the wound gap was observed and photographed (magnification 10×). (C, E) The percentages of migrating cells were calculated relative to the original gap. (B) Cell invasion was analysed in GBM cells treated with indicated doses of TMZ or Mel alone or their combination for 48 h. Cell invasion was observed and photographed, and the percentages of invading cells (D, F) were calculated. The level of significance was indicated by *p < 0.05, **p < 0.01, ***p < 0.001 and ****p < 0.0001.

We also explored whether Mel can enhance the inhibitory effects of TMZ on glioblastoma cell migration and invasion. Wound healing assays were used to assess the migration rates of U87‐MG and U118‐MG cells. As shown in Figure 3A, Mel alone inhibited cell migration, but the combination of Mel and TMZ significantly enhanced this inhibition. In the control group, after 48 h, the gap or wound between the cell layers in both cell lines was completely filled by migrating cells. Quantitative analysis revealed that the combination treatment significantly inhibited cell migration compared to Mel or TMZ alone (Figure 3B,C).

Transwell invasion assays were also performed to evaluate the effect of the combined treatment on the invasiveness of U87‐MG and U118‐MG cells. The invading cells on the underside of the filter were stained and counted. As shown in Figure 3D, the combined treatment of Mel and TMZ further reduced the ability of the cells to penetrate the Matrigel compared to either drug alone. Quantitative analysis confirmed that the combination treatment significantly enhanced the inhibition of glioblastoma cell invasion compared to the individual treatments (Figure 3E,F). These results demonstrate that Mel enhances TMZ's inhibitory effects on cell migration and invasion in glioblastoma cells.

### 3.3 Network Pharmacology Analysis

**FIGURE 4:**
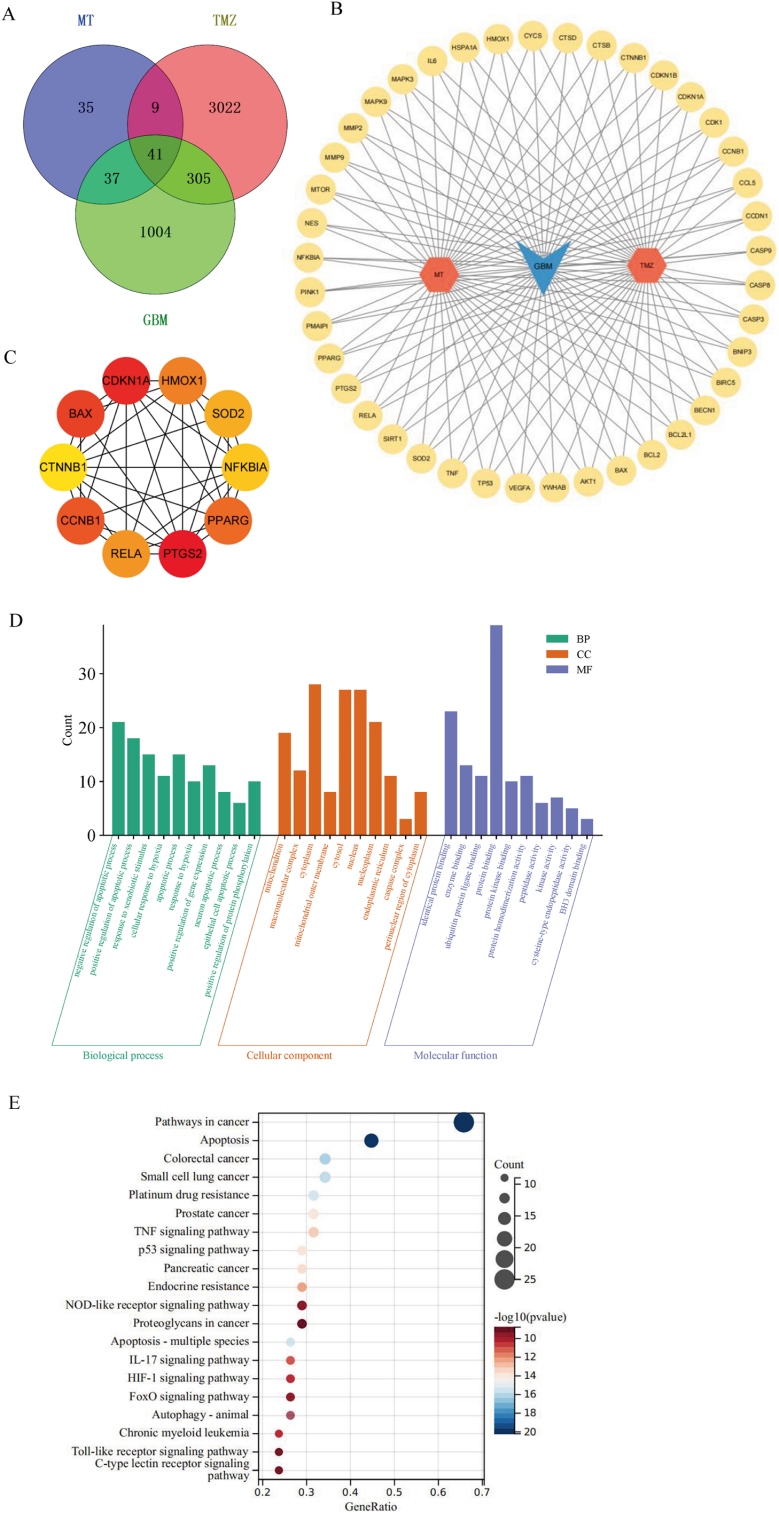
Network pharmacology was used to predict the targets and mechanism of Mel and TMZ in glioma. (A) Venn diagram of common targets of Mel, TMZ and GBM. (B) A network diagram of 41 Mel‐TMZ‐GBM interaction targets. (C) Top 10 hub genes based on the highest DMNC score, analysed by CytoHubba. (D) Gene Ontology (GO) analysis of common targets. BP, biological process; CC, cellular component; MF, molecular function. (E) Kyoto Encyclopedia of Genes and Genomes (KEGG) analysis of common targets.

To uncover the molecular mechanism of Mel's synergistic anti‐glioblastoma effects with TMZ, we utilised network pharmacology to predict the targets and mechanisms of Mel's collaboration with TMZ in treating gliomas. We have collected the relevant targets of Mel, TMZ, and glioblastoma, which are presented in a Venn diagram (Figure 4A). In the diagram, we have identified 122 targets for Mel, 3377 targets for TMZ, and 1387 glioblastoma‐related targets. Among these, 41 common targets were obtained, and a drug‐disease target network was constructed, displaying their gene names (Figure 4B), serving as potential candidate targets for the synergistic treatment of glioblastoma with Mel and TMZ. Furthermore, the top 10 core targets were screened out and presented in Figure 4C and Table 2. These include PTGS2 (COX 2), CDKN1A, BAX, CCNB1, PPARG, HMOX1, RELA, SOD2, NFKBIA and CTNNB1. Among them, PTGS2, also known as COX‐2 ranks first, so we speculate that COX‐2 may play a pivotal role in the synergistic anti‐glioblastoma effect of Mel with TMZ.

**TABLE 2:** Top 10 hub genes.

No.	Name	Score
1	PTGS2	2.374555075
2	CDKN1A	2.311652954
3	BAX	2.309068689
4	CCNB1	2.302939579
5	PPARG	2.295927424
6	HMOX1	2.278610404
7	RELA	2.269320023
8	SOD2	2.20717602
9	NFKBIA	2.200813798
10	CTNNB1	2.180653779

Simultaneously, GO enrichment analysis revealed the molecular functions, cellular components, and biological processes associated with the 41 target genes (Figure 4D). By filtering with a *p*‐value threshold of < 0.05, we obtained significant GO enrichment terms and displayed the top 10 items for each category, as shown in Figure 4D. In the biological processes, we found that they were mainly related to negative regulation of apoptotic process, positive regulation of apoptotic process, response to external stimulus, cellular response to hypoxia, apoptotic process, positive regulation of gene expression, neuronal apoptotic process, epithelial cell apoptotic process and positive regulation of protein phosphorylation. In the cellular components, the most significant enrichments were related to mitochondria, macromolecular complex, cytoplasm, mitochondrial outer membrane, cytosol, nucleus, nucleoplasm, endoplasmic reticulum, caspase complex and perinuclear region of cytoplasm; In the molecular functions, the enrichments were mainly associated with identical protein binding, enzyme binding, ubiquitin protein ligase binding, protein binding, protein homodimerisation activity, peptidase activity, kinase activity, cysteine–type endopeptidase activity and BH3 domain binding. To identify relevant diseases and pathways, a KEGG enrichment analysis was performed. After excluding pathways unrelated to cancer, we selected the top 20 pathways for analysis based on their *p*‐values (Figure 4E). Through the enrichment analysis, it can be discovered that the synergistic effect of Mel and TMZ on glioblastoma is primarily related to apoptosis and TNF signalling pathways. NF‐κB is a key upstream molecule that regulates COX‐2 in the TNF signalling pathway. This regulatory effect is achieved through the activation of IκBα, which induces apoptosis in cells. Therefore, in subsequent studies, we will select relevant proteins for verification.

### 3.4 COX‐2 Is Highly Expressed in the GBM

**FIGURE 5:**
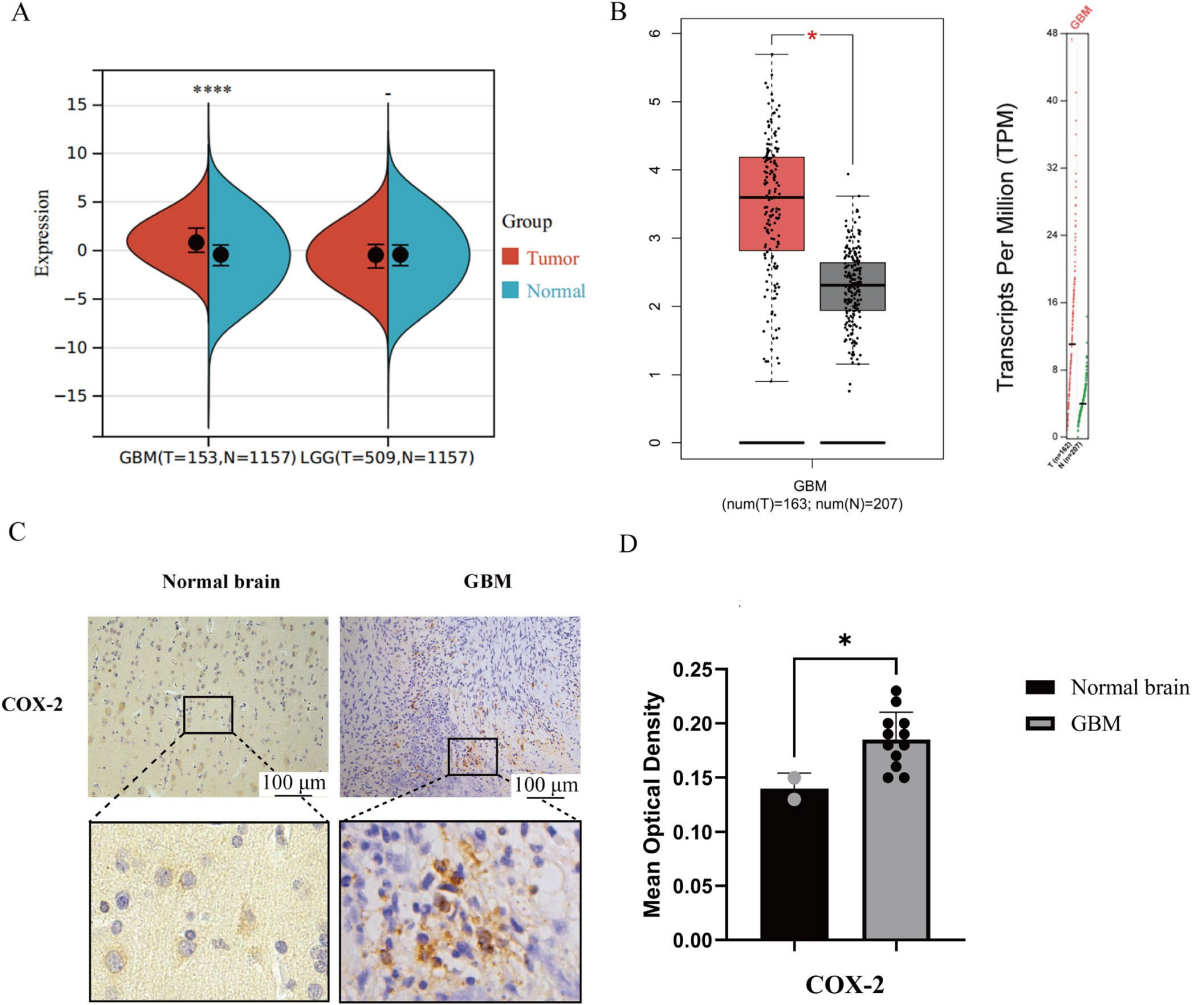
The differential expression of COX‐2 in normal brain tissue and GBM through bioinformatics and immunohistochemistry. (A) The expression difference of COX‐2 in various gliomas and their normal brain tissues. (B) Differential COX‐2 expression within GBM and normal brain tissue. (C) Positive expression of COX‐2 (×200) expression in human GBM and normal brain tissue (magnification 10×). (D) Quantification of COX‐2 expression levels in GBM tissues. Staining intensity was assessed via average optical density (AOD) analysis. Results are presented as mean ± standard deviation (*n* = 12 GBM biopsies; *n* = 2 normal brain specimens). Statistical significance versus normal brain tissue *p < 0.05, and ****p < 0.0001.

Subsequently, we conducted bioinformatics analysis to compare the differential expression of COX‐2 in different grades of gliomas and normal brain tissues. It was found that there was no significant difference in COX‐2 expression between low‐grade gliomas (LGG) and normal brain tissues, but COX‐2 was highly expressed in glioblastomas (GBM). This suggests that the expression level of COX‐2 may be positively correlated with the malignancy of gliomas, as shown in Figure 5A. Meanwhile, we verified the expression of COX‐2 in glioblastomas using the GEPIA website (https://gepia.cancer‐pku.cn/), which further confirmed that the expression of COX‐2 in glioblastomas is higher than that in normal brain tissues, as shown in Figure 5B.

### 3.5 Mel and TMZ Combination Suppressed NF‐κB/COX‐2 Signalling

**FIGURE 6:**
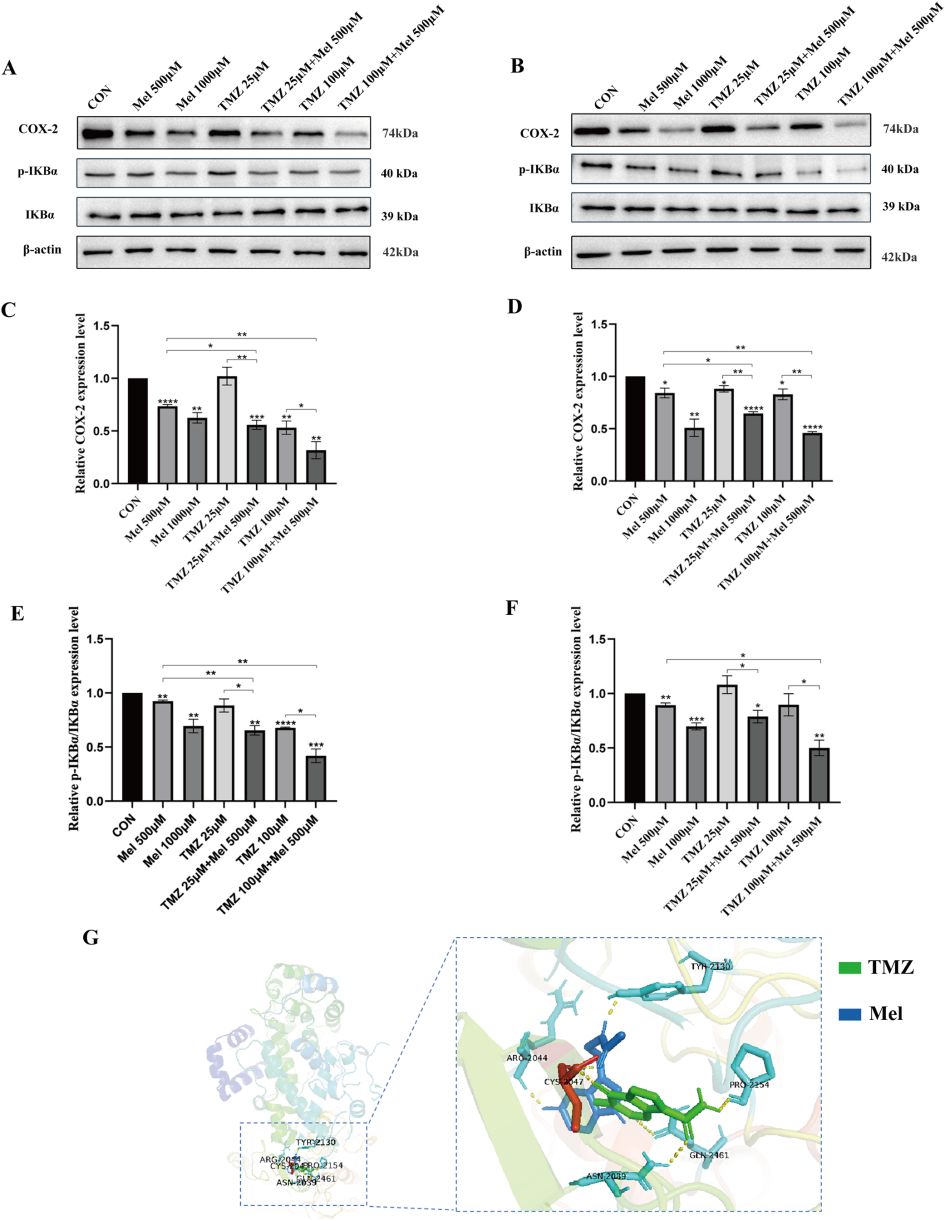
Effect of Mel and TMZ combination on NF‐κB/COX‐2 signalling. U87‐MG cells and U118‐MG cells were treated with Mel (500 μM) or TMZ (25 μM, 100 μM) alone or their combination. (A, B) At 48 h after treatment, the protein levels of COX‐2, IKBα and p‐IKBα in U87‐MG and U118‐MG were detected by western blot analysis. (C) Statistical analysis of COX‐2, IKBα and p‐IKBα protein expression levels in U87‐MG cells by Mel synergistic TMZ. (D) Statistical analysis of COX‐2 protein expression levels in U118‐MG cells by Mel synergistic TMZ. (E) Statistical analysis of IKBα/p‐IKBα protein expression levels in U87‐MG cells by Mel synergistic TMZ. (F) Statistical analysis of IKBα/p‐IKBα protein expression levels in U118‐MG cells by Mel synergistic TMZ. (G) The three‐dimensional molecular docking structure of Mel and TMZ bound to COX‐2. *n* = 3 for each group. Statistical significance was defined as *p < 0.05, **p < 0.01, ***p < 0.001 and ****p < 0.0001.

Increasing evidence suggests that the NF‐κB/COX‐2 pathway played a crucial role in glioblastomas. Therefore, we investigated whether Mel enhances the anti‐glioblastoma effect of TMZ through the NF‐κB/COX‐2 pathway. Molecular docking was used to simulate the interactions between Mel, TMZ and the core target protein COX‐2 (Figure 6G). The results showed that both Mel and TMZ showed positive effects on the active site of COX‐2, and they shared the same binding site. Next, we used Western blotting (WB) to detect the expression levels of NF‐κB/COX‐2 signalling pathway–related proteins such as IκBα, p‐IκBα and COX2 after drug treatment (Figure 6A–E). The results indicated that pretreatment with Mel and TMZ inhibited the expression of COX‐2 and p‐IκBα/IκBα, and the inhibitory effect was stronger when Mel and TMZ were used in combination. These findings suggested that Mel enhanced the anti‐glioblastoma effect of TMZ by inhibiting the NF‐κB/COX‐2 signalling pathway.

### 3.6 Mel and TMZ Combination Promoted Cell Apoptosis

**FIGURE 7:**
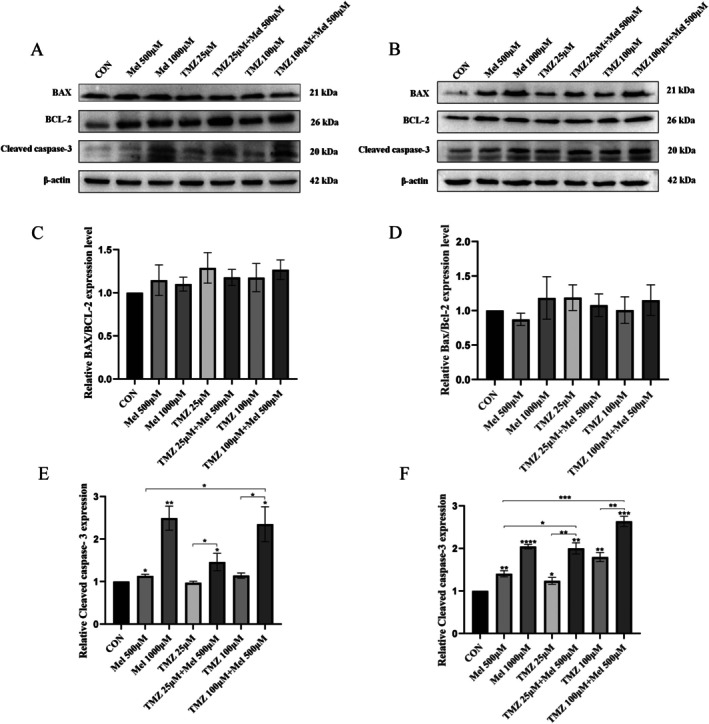
Effect of Mel and TMZ combination on apoptosis‐related proteins. U87‐MG cells and U118‐MG cells were treated with Mel (500 μM) or TMZ (25 μM, 100 μM) alone or in combination. (A) At 48 h after treatment, the protein levels of BAX, Bcl‐2 and Cleaved caspase‐3 in U87‐MG were detected by western blot analysis. (B) At 48 h after treatment, the protein levels of BAX, Bcl‐2, and Cleaved caspase‐3 in U118‐MG were detected by western blot analysis. (C) Statistical analysis of BAX/Bcl‐2 protein expression levels in U87‐MG cells by Mel synergistic TMZ. (D) Statistical analysis of BAX/Bcl‐2 protein expression levels in U118‐MG cells by Mel synergistic TMZ. (E) Statistical analysis of Cleaved caspase‐3 protein expression levels in U87‐MG cells by Mel synergistic TMZ. (F) Statistical analysis of Cleaved caspase‐3 protein expression levels in U118‐MG cells by Mel synergistic TMZ. *n* = 3 for each group. Statistical significance was defined as *p < 0.05, **p < 0.01, ***p < 0.001 and ****p < 0.0001.

To elucidate the effects of the drugs on the apoptotic signalling pathway in glioblastomas, Western Blot analysis was performed to detect the protein expression levels of apoptosis‐related proteins BAX and BCL‐2, and Cleaved caspase‐3 in U87‐MG and U118‐MG cells after 48 h of drug treatment. As shown in Figure 7A,B, after 48 h of treatment with Mel at concentrations of 500 and 1000 μM, the expression of Cleaved caspase‐3 was significantly increased compared to the untreated cells, and the difference was statistically significant (*p* < 0.05); the expression level of Cleaved caspase‐3 was increased when 500 μM Mel was combined with 25 μM TMZ or 100 μM TMZ, compared to the use of Mel or TMZ alone, and the difference was statistically significant (*p* < 0.05). However, although both BAX and BCL‐2 showed varying degrees of increase after treatment with the drugs in each group, the ratio of BAX to BCL‐2 did not show significant changes (*p* > 0.05). These results suggested that Mel synergised with TMZ to induce apoptosis in glioblastoma cells through a non‐mitochondrial apoptotic pathway.

### 3.7 The Mitochondrial Membrane Potential Remained Unchanged After Mel Treatment

To assess Δψm in U118‐MG cells following drug treatment, we performed JC‐1 staining and visualised cells using a fluorescence microscope. The JC‐1‐treated cells emitted red fluorescence, with no detectable green fluorescence, indicating that the Δψm remained intact and was not disrupted by Mel treatment (Figure S2).

### 3.8 Therapeutic Efficacy of Mel Combined With Temozolomide in GL261 Subcutaneous Xenograft Mouse Model

**FIGURE 8:**
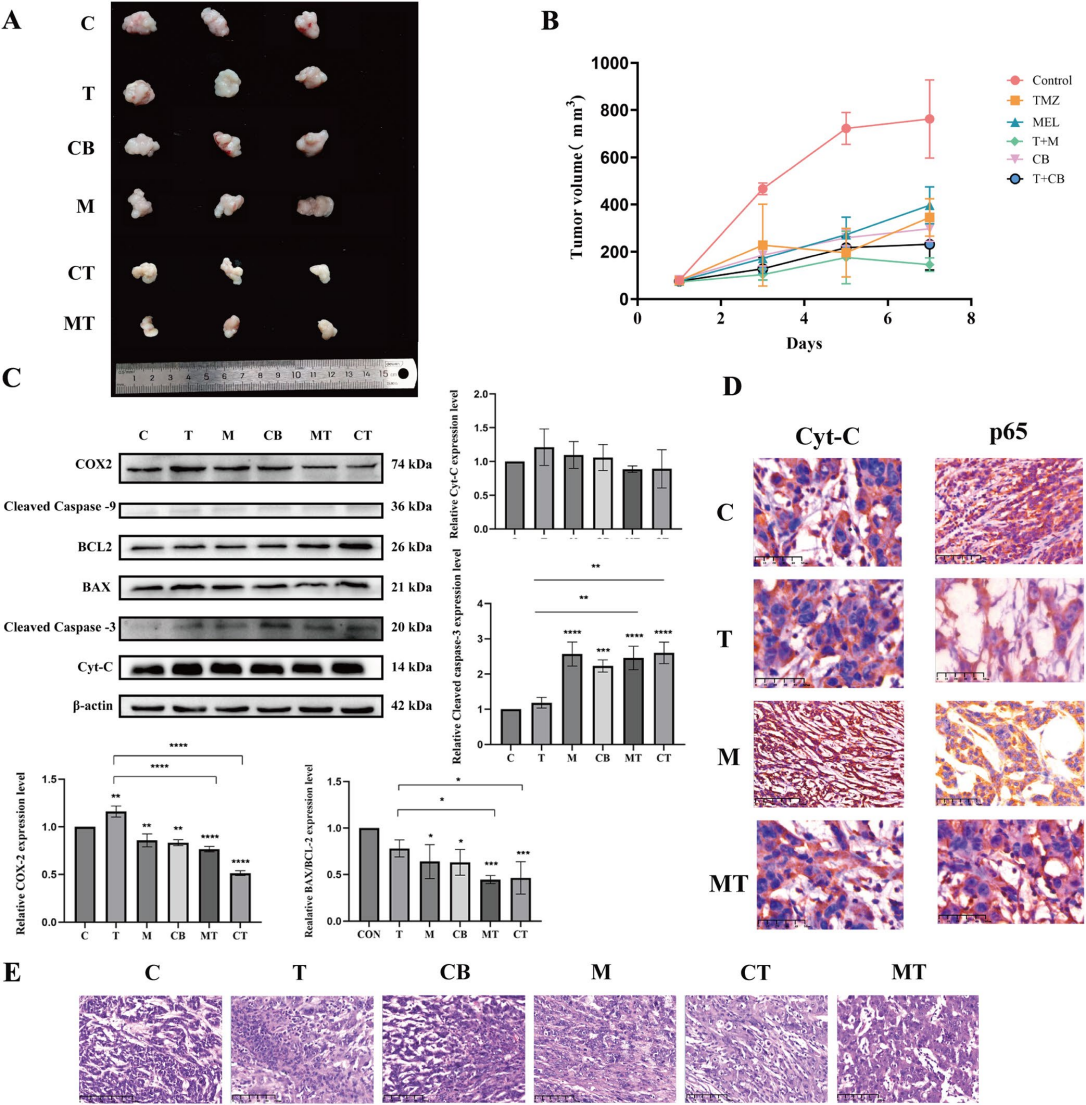
Research results on the therapeutic efficacy of Mel combined with TMZ in the GL261 subcutaneous xenograft mouse model. (A) Representative images of tumour. (B) Changes in tumour volume. (C) Expression profiles of apoptosis‐related proteins and COX2 protein in subcutaneous xenograft tumours. (D) Immunohistochemical localization images of p65 and Cyt‐C expression in xenograft tumours (magnification 4×). (E) HE staining of xenograft tumours. All data are presented as the mean ± SD derived from seven individual fields across six independent experimental replicates per treatment group (magnification 40×). (*p < 0.05, **p < 0.01, ***p < 0.001, ****p < 0.0001 vs. control group).

To investigate the potential clinical relevance of co‐administering Mel and TMZ in glioblastoma treatment, we established a GL261 subcutaneous xenograft model in mice. Tumour growth inhibition was evaluated in vivo, and the selective COX‐2 inhibitor celecoxib was used to explore the potential involvement of COX‐2 in the observed synergistic effects. As shown in Figure 8A, combined treatment with Mel and TMZ significantly inhibited tumour growth in vivo. Tumour volume at the end of treatment was significantly reduced in the combination group compared to the control group (Figure 8B).

Haematoxylin and eosin (HE) staining of tumour sections revealed extensive necrotic areas in the Mel + TMZ group (Figure 8E). These regions exhibited features such as nuclear pyknosis and lysis, eosinophilic cytoplasm and clear demarcation from viable tumour regions, where cells were tightly packed, exhibited more regular morphology, and showed reduced nuclear‐to‐cytoplasmic ratios along with stromal fibrosis–indicating markedly suppressed tumour proliferation. Notably, celecoxib alone or in combination with TMZ produced tumour‐suppressive effected similar to those observed in the Mel or Mel + TMZ groups.

Western blot analysis demonstrated that Mel in combination with TMZ significantly downregulated COX‐2 expression (*p* < 0.05), consistent with the in vitro findings. Likewise, celecoxib alone or combined with TMZ exhibited comparable COX‐2 suppression (Figure 8C).

To further explore the involvement of apoptosis‐related pathways, we assessed the expression of BAX, BCL‐2, Cleaved caspase‐3 and caspase‐9. Cleaved caspase‐3 levels were significantly elevated following Mel treatment alone or in combination with TMZ (*p* < 0.05). However, the BAX/BCL‐2 ratio did not significantly increase, and only weak Cleaved caspase‐9 signals were detected, with levels far below that of Cleaved caspase‐3. Cyt‐C expression exhibited no statistically significant differences across groups (*p* > 0.05). These results collectively suggested that Mel‐induced apoptosis, either alone or in combination with TMZ, was not mediated through the classical mitochondrial apoptotic pathway.

Immunohistochemistry revealed that p65 localisation was predominantly cytoplasmic in tumour cells from the Mel and Mel + TMZ groups, aligning with the in vitro findings that Mel enhances TMZ efficacy by inhibiting the NF‐κB/COX‐2 signalling pathway. Furthermore, immunohistochemical staining of Cyt‐C in tumour tissues from the control, TMZ, Mel, and TMZ + Mel groups revealed a consistent staining pattern across all groups: Cyt‐C was primarily localised within mitochondria, appearing as punctate, dark brown staining surrounding the nucleus, indicative of strong mitochondrial localisation. In contrast, cytoplasmic staining was weak or absent. This mitochondrial–restricted localisation of Cyt‐C suggested that under all tested conditions, the mitochondrial apoptotic pathway was not activated and that Cyt‐C remained engaged in its physiological role in mitochondrial respiration rather than in apoptotic signalling (Figure 8D).

## 4 Discussion

Glioblastoma (GBM) is a highly aggressive malignant tumour of the central nervous system, accounting for 49.1% of all primary malignant tumours of the central nervous system [35]. The incidence of GBM is relatively high, with about 3.2 new cases per 100,000 people each year, and it is expected to continue to rise over the next 30 years, posing a significant challenge to global public health [36, 37]. Despite the median survival period of patients with GBM typically being around 12 months, only a few patients can survive beyond 5 years [38]. Since 2005, the 26,981 trial conducted by the European Organisation for Research and Treatment of Cancer has brought significant breakthroughs in the treatment of GBM. The results of this trial showed that the treatment regimen combining TMZ with radiotherapy (known as the Stupp protocol) significantly improved the survival rate of newly diagnosed GBM patients compared to traditional radiotherapy alone. As a result, the Stupp protocol has been recommended in both domestic and international guidelines as thefirst‐line treatment for patients with GBM, firmly establishing the primary status of TMZ among chemotherapy drugs for GBM [39]. However, despite the significant efficacy of TMZ, its therapeutic effect is still limited by decreased drug sensitivity and toxic side effects, resulting in unsatisfactory treatment outcomes. Therefore, the high recurrence rate and mortality of GBM multiforme, as well as the high resistance rate to the first‐line chemotherapeutic drug TMZ, urge researchers to continually explore more effective treatment methods.

Mel, an endogenous indoleamine, can safely and efficiently cross the blood–brain barrier to exert its effects [40]. Multiple experiments have confirmed the synergistic effect of Mel with various chemotherapeutic drugs, demonstrating its ability to reduce toxicity and enhance efficacy in both in vitro and in vivo experiments [41]. The combined application of Mel and cisplatin can promote apoptosis in the human ovarian cancer cell line OVCAR3 and the osteosarcoma cell line MG63 by inhibiting the PI3K/Akt signalling pathway [42, 43]. Mel significantly reverses cisplatin resistance in cisplatin‐resistant cancer cell lines. In nasopharyngeal carcinoma, Mel enhances the sensitivity of cancer cells to cisplatin by inhibiting the Wnt/β‐catenin signalling pathway [44]. Additionally, in hepatocellular carcinoma, Mel enhances the chemosensitivity of cancer cells to cisplatin by regulating the AP‐2β/hTERT and NF‐κB/COX‐2 signalling pathways [45]. In vivo experiments, researchers found that the combined administration of Mel and doxorubicin (Dox) reduced Dox‐induced neuronal degeneration in the hippocampus and prefrontal cortex. This neuroprotective effect was mediated by enhancing endogenous antioxidants and activating the Nrf2/p53‐SIRT1 signalling pathway in the prefrontal cortex of adult rats, thereby promoting neurogenesis and mitigating Dox‐induced neurotoxicity [46]. Both in vivo and in vitro studies have demonstrated that Mel effectively inhibits the CDDP‐induced upregulation of Bax, p53, and AQP3, thereby alleviating cardiac edema associated with CDDP treatment [47]. Additionally, researchers have found that Mel effectively protects reproductive–age female patients from CDDP‐induced ovarian damage [48]. Furthermore, experimental validation has confirmed that Mel exerts protective effects against CDDP‐induced acute kidney injury [49, 50] and testicular damage [51]. Clinical trials have further revealed that Mel can not only alleviate the side effects of chemotherapy or radiotherapy for patients but also potentially improve the treatment outcomes for advanced cancer patients with poor clinical conditions [52]. However, whether Mel can enhance the therapeutic effect of TMZ in GBM remains to be further investigated.

In this study, we assessed the response of GBM to the combination of Mel and TMZ. Using the Chou–Talalay method to calculate the combination index (CI), we found a synergistic effect between Mel and TMZ in the U87‐MG and U118‐MG GBM cell lines. When used in combination, the two drugs exhibited stronger inhibitory effects on both cell lines compared to either drug alone. Furthermore, cell experiments showed that the combination of Mel and TMZ enhanced the inhibition of GBM cell proliferation, migration, and invasion. Nuclear factor‐κB (NF‐κB) is a key transcription factor that plays a central role in tumours, involving multiple aspects such as proliferation, invasion, metastasis and apoptosis [53]. In GBM, NF‐κB is abnormally activated, which helps cancer cells evade apoptosis and resist the treatment of chemotherapy drugs [54]. Moreover, COX‐2 is frequently overexpressed in GBM, and its aberrant activation is closely linked to increased tumour aggressiveness and chemoresistance [39, 55, 56]. Previous studies have shown that Mel can downregulate COX‐2 expression through inhibition of the NF‐κB signalling pathway, exerting anti‐inflammatory and antitumour effects [57]. In early glioma models, Mel significantly reduced COX‐2 levels in rat C6 glioma cells, suggesting a neuroprotective role in glial cells [58] In neural stem cell systems, Mel modulates inflammation and neurogenesis by suppressing COX‐2 and prostaglandin synthesis [59]. In other cancer types, such as breast cancer, Mel has been reported to regulate several signalling pathways, including COX‐2, p300, Akt and Apaf‐1, thereby inhibiting cell proliferation and promoting apoptosis [60]. Furthermore, Mel modulates TLR4‐mediated inflammatory gene expression via both MyD88‐ and TRIF‐dependent pathways, highlighting its broader role in immune regulation [61]. Its derivatives have also demonstrated COX‐2‐suppressing and antitumour properties across various tumour models.

While these findings provide a strong foundation for the role of Mel in regulating COX‐2‐related signalling, the potential of Mel as a chemosensitiser in GBM—especially in TMZ‐resistant settings—has not been thoroughly explored. Our study builds on this knowledge by examining the application of Mel in a TMZ‐based therapeutic effect in GBM. We systematically demonstrated that Mel enhances the antitumour efficacy of TMZ by suppressing the NF‐κB/COX‐2 signalling cascade. Both in vitro and in vivo results confirmed that Mel effectively downregulates COX‐2 expression, prevents nuclear translocation of p65, and partially overcomes TMZ resistance. This suggests that this signalling axis could be a promising target for improving GBM chemosensitivity.

Thus, this study not only expands on previous work regarding Mel‐mediated COX‐2 regulation but also offers novel mechanistic insights and a translational rationale for its potential in overcoming chemoresistance in GBM. Our results indicate that the combination of Mel and TMZ produces a synergistic antitumor effect in GBM, primarily through suppression of theNF‐κB/COX‐2 signalling pathway and activation of caspase‐dependent apoptosis. Network pharmacology analysis initially identified COX‐2 as a key therapeutic target in the synergy. Subsequent bioinformatics analyses revealed that COX‐2 was significantly overexpressed in GBM compared to both low‐grade gliomas and normal brain tissues, correlating with tumour aggressiveness. This was further validated using the GEPIA database and immunohistochemistry of patient samples, both of which confirmed high COX‐2 expression levels in GBM tissue. These data supported COX‐2 as a clinically and mechanistically relevant target in GBM progression.

To explore this mechanism in vivo, a subcutaneous xenograft model was established, and a selective COX‐2 inhibitor, celecoxib, was included to assess whether the observed synergy was mediated via COX‐2 suppression. Results showed that Mel + TMZ significantly suppressed tumour growth compared to monotherapy, and the degree of tumour inhibition was comparable to that of Celecoxib + TMZ, suggesting COX‐2 as a critical mediator of the combinatorial efficacy. Western blot analysis confirmed that Mel alone or in combination with TMZ downregulated COX‐2 expression in tumour tissues. Furthermore, immunohistochemical staining revealed that p65, a key NF‐κB subunit, remained sequestered in the cytoplasm without clear nuclear translocation, supporting the inhibition of NF‐κB activity observed at the protein level.

Molecular docking analysis further confirmed that Mel and TMZ share a common binding site on COX‐2 with favourable binding affinity, suggesting that the combination may enhance pharmacological engagement with the COX‐2 target, leading to downstream suppression of inflammatory and pro‐survival signalling.

Apoptosis plays a central role in the anti‐tumour response, with key regulators such as BAX, BCL‐2, and Cleaved caspase‐3 often being evaluated in mitochondrial apoptosis way [62, 63, 64]. In this study, although Mel and TMZ influenced the expression of BAX and BCL‐2, their ratio (BAX/BCL‐2) remained largely unchanged following treatment. In addition, Cleaved caspase‐9 was undetectable, and JC‐1 staining showed no significant loss of mitochondrial membrane potential, further indicating apoptosis proceeded via a mitochondria‐independent route.

Emerging evidence suggests that Mel can activate non‐canonical apoptotic pathways, including the Fas/FasL axis, p53 upregulation, ER stress, and oxidative stress. These pathways all converge on the activation of Caspase‐8 and the subsequent cleavage of Caspase‐3. This mechanism provides a plausible explanation for the activation of Caspase‐3 in the absence of Caspase‐9 cleavage and Cyt‐C release [65]. Additionally, some studies have proposed that Mel may be metabolised by CYP1B1 within tumour mitochondria, producing N‐acetylserotonin (NAS). This, in turn, activates the CYP1B1‐NAS‐Cyt‐C signalling axis [66]. On the other hand, TMZ has been shown to alter mitochondrial dynamics by promoting mitochondrial fusion, which is associated with chemoresistance. By promoting mitochondrial fission, Mel may counteract this fusion‐driven chemoresistance, increase reactive oxygen species (ROS) levels, and thereby enhance the susceptibility of tumour cells to apoptosis [67].

**FIGURE 9:**
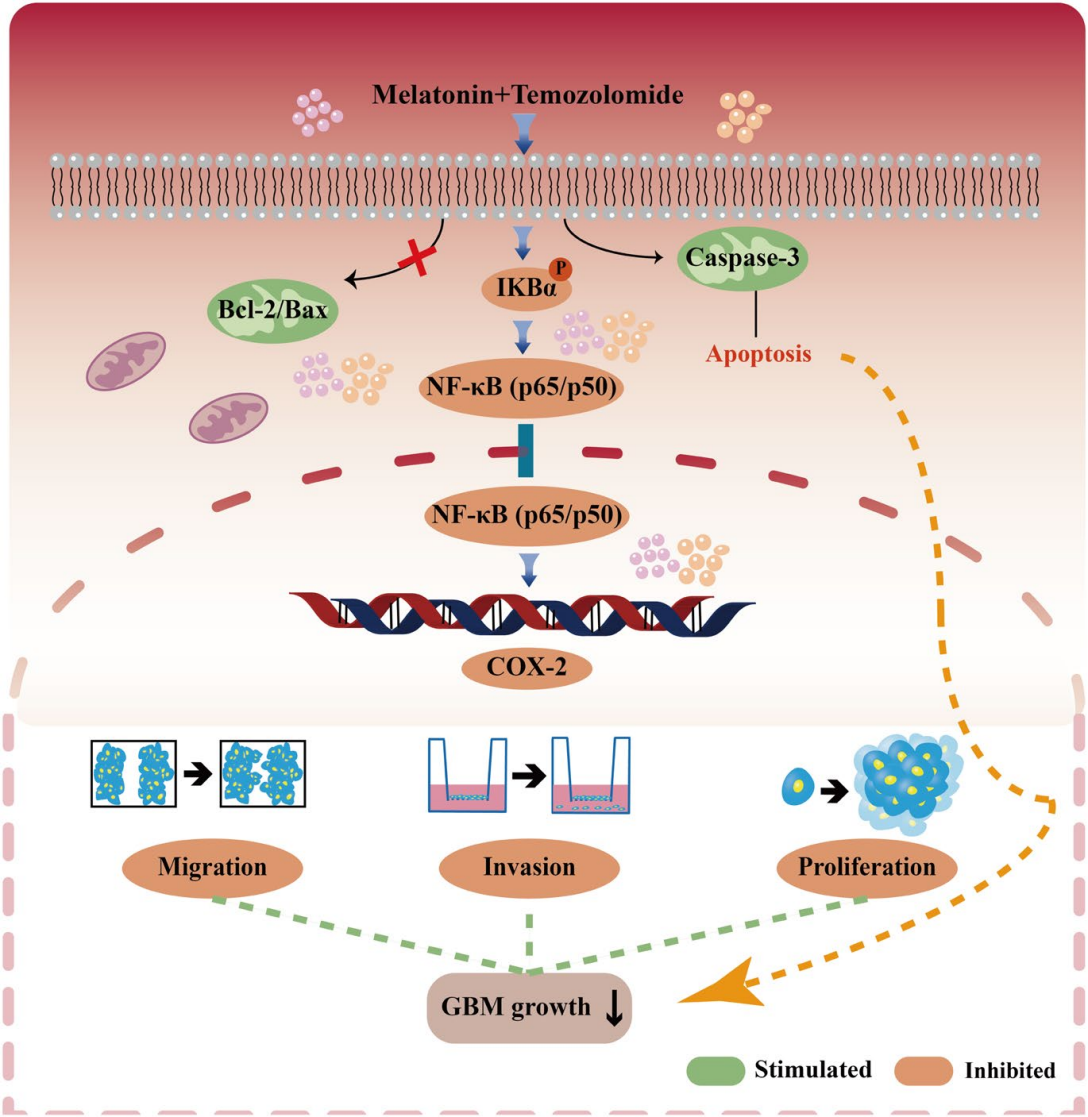
The mechanism of the action of Mel combined with TMZ in GBM cells.

In conclusion, our findings indicate that Mel enhances the anti‐GBM efficacy of TMZ through dual mechanisms: inhibition of the NF‐κB/COX‐2 signalling axis and induction of caspase‐3‐mediated apoptosis via a mitochondria‐independent pathway (Figure 9). These results provide novel mechanistic insight into the combinatorial potential of Mel + TMZ and support its further evaluation as a promising therapeutic strategy for GBM.

## Author Contributions

**Hong Tang:** conceptualization (equal), formal analysis (equal), writing – original draft (equal). **Qi Dai:** formal analysis (equal), writing – review and editing (equal). **Ziyan Zhao:** conceptualization (equal), formal analysis (equal). **Weiye Ge:** data curation (equal), formal analysis (equal). **Danlei Li:** data curation (equal), formal analysis (equal). **Zelin Chang:** data curation (equal), formal analysis (equal). **Penglai Pi:** data curation (equal), formal analysis (equal). **Jia Li:** conceptualization (lead), project administration (lead), supervision (lead). **Zheng Sun:** conceptualization (lead), project administration (lead), supervision (lead), writing – review and editing (equal).

## Ethics Statement

Our study was conducted with the approval of the Ethics Committee of the First Affiliated Hospital of Dalian Medical University.

## Conflicts of Interest

The authors declare no conflicts of interest.

## Supporting information


**Figure S1:** The effects of treatment with Mel, TMZ, or their combination, as well as CB, TMZ alone, or their combination at the indicated dose, on cell viability and proliferation in GL261 cells were evaluated. After 48 h treatment, cell viability was assessed using the MTT assay. The cell viability of the Mel (500 μM group) was set as 100% reference. Data were presented as the mean ± standard deviation (SD) from three independent experiments. Statistical significance was indicated as ****p < 0.0001.

**Figure S2:** Detection of mitochondria membrane potential (Δψm) using JC‐1 (magnification 20×). U118MG cells exhibited no detectable green fluorescence signal following treatment with TMZ and/or Mel (alone or in combination).

## Data Availability

The Western blot, immunohistochemistry, and other related experimental data generated and analyzed during the current study are included within this published article and its Supporting Information files. Additional data are available from the corresponding author upon reasonable request.
