# Supplementary material for: Melatonin Synergises the Chemotherapeutic Effect of Temozolomide in Glioblastoma by Suppressing NF‐κB/COX‐2 Signalling Pathways
Source: J Cell Mol Med. 2025 Aug 13;29(15):e70778. doi: 10.1111/jcmm.70778 (PMC12350191; doi:10.1111/jcmm.70778)
Supplement: Supplementary file 1 — Figure S1: The effects of treatment with Mel, TMZ, or their combination, as well as CB, TMZ alone, or their combination at the indicated dose, on cell viability and proliferation in GL261 cells were evaluated. After 48 h treatment, cell viability was assessed using the MTT assay. The cell viability of the Mel (500 μM group) was set as 100% reference. Data were presented as the mean ± standard deviation (SD) from three independent experiments. Statistical significance was indicated as ****p < 0.0001. [file JCMM-29-e70778-s001.docx]

**Supplementary Figure1**


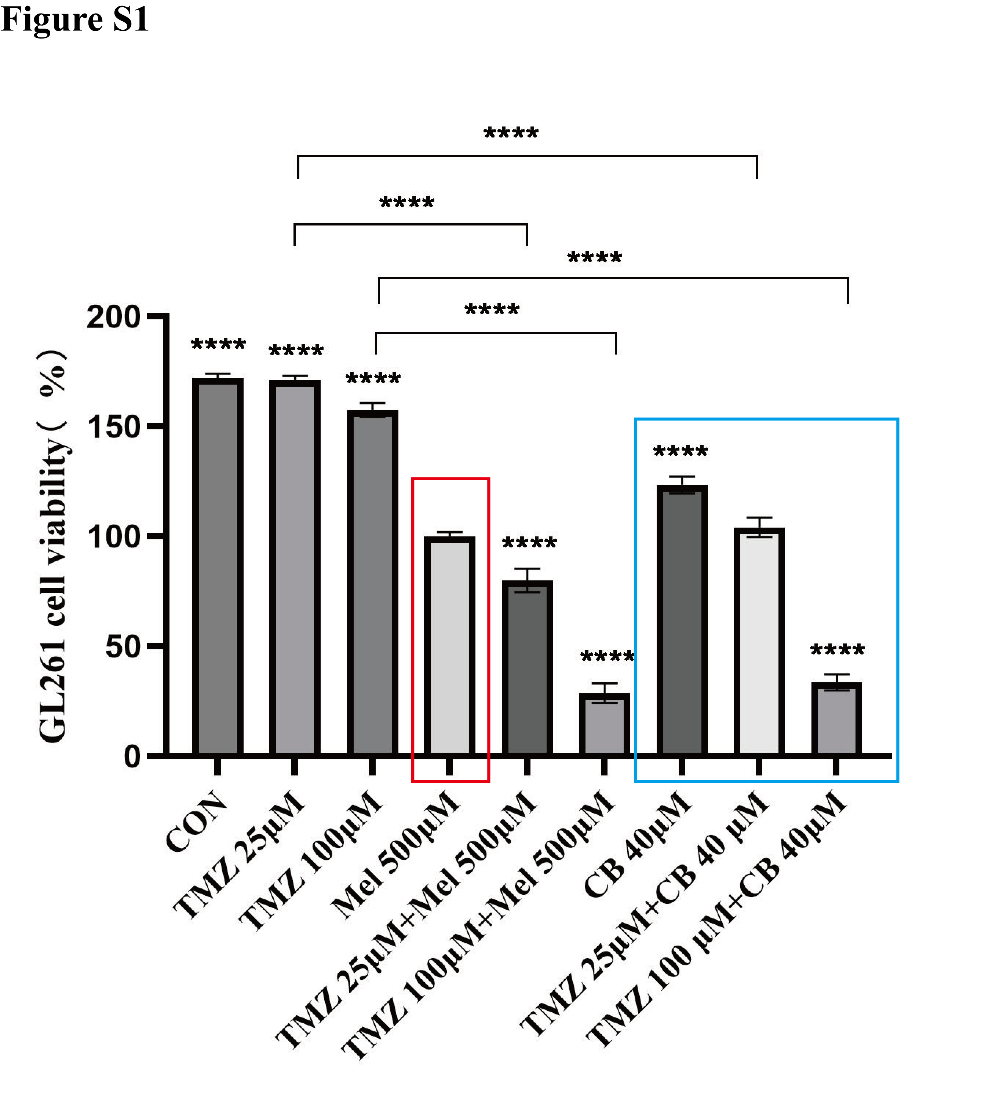


**Figure S1.** The effects of treatment with Mel, TMZ, or their combination, as well as CB, TMZ alone, or their combination at the indicated dose, on cell viability and proliferation in GL261 cells were evaluated. After 48 hours treatment, cell viability was assessed using the MTT assay. The cell viability of the Mel (500 μM group) was set as 100% reference. Data are presented as the mean ± standard deviation (SD) from three independent experiments. Statistical significance is indicated as *****P* < 0.0001.
