# Supplementary material for: Melatonin Synergises the Chemotherapeutic Effect of Temozolomide in Glioblastoma by Suppressing NF‐κB/COX‐2 Signalling Pathways
Source: J Cell Mol Med. 2025 Aug 13;29(15):e70778. doi: 10.1111/jcmm.70778 (PMC12350191; doi:10.1111/jcmm.70778)
Supplement: Supplementary file 2 — Figure S2: Detection of mitochondria membrane potential (Δψm) using JC‐1 (magnification 20×). U118MG cells exhibited no detectable green fluorescence signal following treatment with TMZ and/or Mel (alone or in combination). [file JCMM-29-e70778-s002.docx]

**Supplementary Figure2**

**
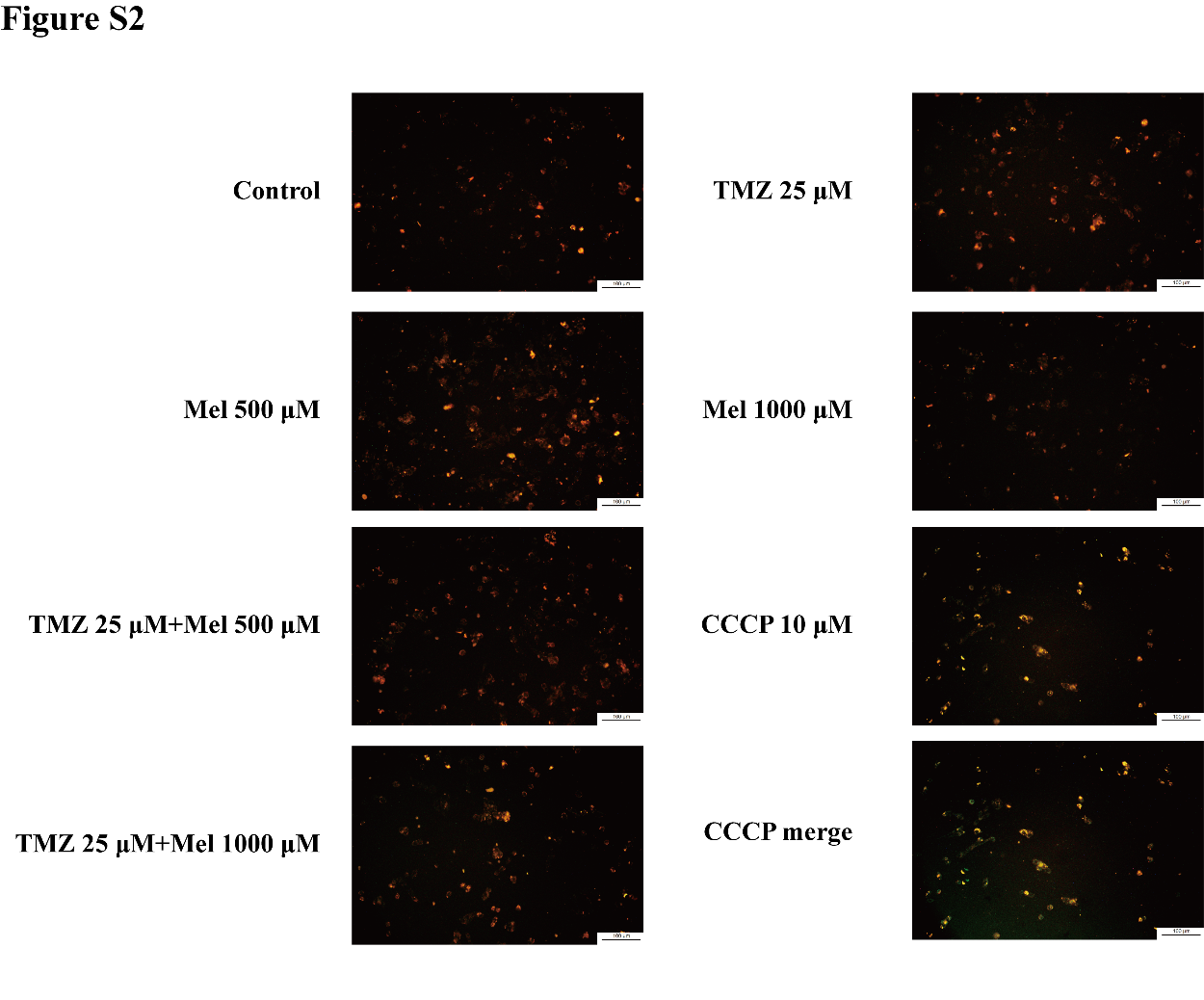
 Figure S2.** Detection of mitochondria membrane potential (Δψm) using JC-1 (magnification 20×). U118MG cells exhibited no detectable green fluorescence signal following treatment with TMZ and/or Mel (alone or in combination).
